# Pancreatic surgery during the COVID-19 pandemic 2020–2021: an observational cohort study from a third level referral center

**DOI:** 10.1186/s12893-022-01651-7

**Published:** 2022-05-21

**Authors:** Carsten Palnæs Hansen, Jan Henrik Storkholm, Martin Hylleholt Sillesen, Paul Suno Krohn, Stefan Kobbelgaard Burgdorf, Jens Georg Hillingsø

**Affiliations:** Department of Surgery and Transplantation, Rigshospitalet Copenhagen University Hospital, 2100 Copenhagen, Denmark

**Keywords:** COVID, Lockdown effect, Pancreatic surgery, Volume, Waiting time

## Abstract

**Background:**

During the COVID pandemic there has been limited access to elective surgery including oncologic surgery in several countries world-wide. The aim of this study was to investigate if there was any lockdown effect on pancreatic surgery with special focus on malignant pancreatic and periampullary tumours.

**Methods:**

Patients who underwent pancreatic surgery during the two Danish lockdown periods from 11. March 2020 and the following 12 months were compared with patients who were operated the preceding 3 years. Data on patients’ characteristics, waiting time, operations, and clinical outcomes were evaluated.

**Results:**

During lockdown and the previous three years the annual number of resections were 242, 232, 253, and 254, respectively (p = 0.851). Although the numbers were not significantly different, there were fluctuations in operations and waiting time during the lockdown. During the second outbreak of COVID October 2020 to March 2021 the overall median waiting time increased to 33 days (quartiles 26;39) compared to 23 (17;33) days during the first outbreak from March to May 2020 (p = 0.019). The same difference was seen for patients with malignant tumours, 30 (23;36) vs. 22 (18;30) months (p = 0.001). However, the fluctuations and waiting time during lockdown was like the preceding three years. Neither 30- nor 90-days mortality, length of stay, number of extended operations, and complications and tumour stage were significantly different from previous years.

**Conclusions:**

There were significant fluctuations in waiting time for operations during the lockdown, but these variations were not different from the preceding three years, wherefore other explanations than an impact from COVID are conceivable.

## Background

In early 2020 COVID started its progress in Denmark, and on 11. March 2020, COVID was declared a pandemic by the World Health Organization [1]. This day, a state of emergency was implemented in Denmark. Retail trade, restaurants, schools, and higher educations were closed, sporting games and public assemblies were banned, and face masks in public transportation and in food stores became mandatory. After the end of the first outbreak in the middle of April, there was a controlled, gradual re-opening of the country with re-implementation of the restrictions during the second outbreak from November to March which remained in force until 18. May 2021, when they were gradually lifted and finally ended 1. September 2021.

The health system became National priority with direct control by the Prime Minister and her office. Plans were made for the containment of the disease, and special units at regional hospitals were set up to receive patients with COVID. These precautions conformed to the handling in other European countries. However, the way each country re-organized their hospitals differed according to the numbers of needed admission and the pre-COVID resources.

In several countries, health infrastructure was profoundly changed, and at hospitals the surgical departments were either closed or the admissions for elective surgery was dramatically reduced to allocate resources to departments with COVID patients. In countries with the highest disease impact, this resulted in delayed diagnosis and surgical treatment of cancer patients and referral to oncologic treatment instead [2–5]. Moreover, cancer patients apparently run an increased hazard if infected with COVID before as well as after surgical or oncological treatment [6, 7]. Studies are waiting to give a better understanding of the different outcome of oncological surgery during the COVID crisis [8].

Ductal adenocarcinoma of the pancreas (PDAC) has become one of the leading causes of cancer-related deaths world-wide, and due to its rapid progressive nature, diagnosis and treatment has high priority. A survey from 37 countries on five continents reported a reduced number of pancreatic resections during the COVID pandemic, although more than 80% of the responders, most of them surgeons, in a questionnaire strongly advocated for active treatment [9].

Denmark implemented the so-called “Cancer Patient Pathway” in 2008/2009, which aims to ensure that patients suspected of malignant tumors will have a timelier diagnosis and treatment. During lockdown, the Danish Health Authority enjoined the hospitals to continue follow the Cancer Patient Pathway. The outcome of the treatment of patients with PDAC are still pending.

The aim of this study is to investigate the impact of the COVID pandemic on the surgical treatment of patients with pancreatic and periampullary tumours, malignant as well as benign, in a specialized Hepato-Pancreato-Biliary (HPB). The data were compared with the results from the preceding three years before the lockdown to assess for any difference in admission, severity of presentation and outcome.

## Methods

### Patients

Data were divided into two cohorts. One included all patients who underwent pancreatic surgery irrespective of pathology during first year of COVID from 11. March 2020 to 10. March 2021. The results were compared with data from the second cohort of patients operated 11. March 2017 to 10. March 2020. All patients were operated at the HPB unit at Copenhagen University Hospital, Rigshospitalet, which is a tertiary hospital with a catchment area of 2.8 million residents for HPB surgery. The unit is the largest of the four hospitals that in a centralized setting performs HPB surgery in Denmark. The hospital also receives severe cases of COVID in a specialized unit.

### Data collection

Data were collected from the department’s prospectively maintained database of pancreatic operations, from the electronic hospital record systems Orbit and EPIC, the Danish National Pathology Data Registry, and from the National Register of Death. All Danish Nationals have a unique Central Person Registration number that enables searching of health data. The Danish health care registries are complete, and the risk of data loss within the searched fields is minimal [10].

Diseases included adenocarcinoma of the pancreas, common bile duct and the periampullary region, neuroendocrine tumors (NET), non-malignant tumors, intraductal papillary mucinous neoplasia (IPMN) and pancreatitis. Diagnosis was classified according to the International Classification of Diseases ICD-10 (C.17.0, C24.0, C24.1, C25, C25.4, D13.6, D13.7, D37.7 and K86).

### Surgical treatment

The day before admission for pancreatic surgery, all patients were tested for COVID (PCR test) and any time during hospitalization in case of symptoms. In general, relatives were not allowed access to the hospital and only after a negative PCR test. Contact with relatives at the doctor’s round were by mobile phone. All operations before and during lockdown were performed by the same surgical team (four consultants and one staff specialist). Robotic surgery was only used in selected cases of distal pancreatectomy after September 2019. After pancreaticoduodenectomy and total pancreatectomy, but not distal pancreaticoduodenectomy, patients stayed overnight in a high dependency unit.

Resectability of PDAC followed the criteria and guidelines of the International Association of Pancreatology (IAP)/European Pancreatic Club (EPC) [11]. The standard treatment for resectable PDAC in Denmark is upfront surgery without neoadjuvant chemotherapy. In case of locally advanced PDAC, neoadjuvant chemo- or chemoradiotherapy is used according to evaluation at the multidisciplinary team conference (MDT).

Waiting time to operation was defined as time from the presentation at the MDT, where operation was decided, till operation was performed. Length of hospital stay (LOS) was defined as time from operation to discharge with exclusion of in-hospital deaths. Perioperative death was defined as death within 90 days of pancreatic resection.

Patients’ physical status and comorbidity were classified according to the American Society of Anesthesiologists (ASA) and the Royal College of Surgeons Charlson score. The staging of adenocarcinomas followed the American Joint Committee on Cancer, eighth edition, and for NET the WHO Classification of Neuroendocrine Tumors 2019. Surgical complications were classified according to Clavien and Dindo [12].

The primary outcome was waiting time to operation, number and type of operations, and stage of adenocarcinomas. Secondary outcome was LOS, severe surgical complications (leakage from the pancreatic, bile or gastro-jejunal anastomosis, intraabdominal haemorrhage, intraabdominal abscess, organ infarction), perioperative death, and for adenocarcinoma referral to adjuvant chemotherapy.

### Statistical analysis

The study is an observational cohort study and reported according to the STROCSS guidelines [13]. Data are presented as median and interquartile range or mean when appropriate and categorial data as numbers and percentage. Analysis of contingency tables was performed by Fisher’s exact test. Data between subgroups were analyzed with the Mann–Whitney and Kolmogorov–Smirnov tests. Ordinary and repeated measures of variance were performed by the Kruskal–Wallis and Friedman tests. Statistical significance was considered as 2-tailed p*-*value < 0.05 for all tests. Analysis was performed with GraphPad Prism software version 9.2.0 (GraphPad, La Jolla, CA).

## Results

Patients’ characteristics before and during lockdown were not significantly different (Table 1).

**Table 1:** Characteristics and clinical outcome of patients undergoing pancreatic surgery before and during the COVID lockdown period.Ordinal data presented as median (25;75 quartiles)

	Before lockdown					
	2017/2018	2018/2019	2019/2020	Total	Lockdown	*p* value
Numbers	254	253	232	739	242	0.851
Males/females	153/101	137/116	123/109	415/326	140/102	
Age, years	69 (62;73)	67 (58;73)	69 (61;74)	68 (60;73)	68 (59;75)	0.140
*ASA (%)*						
1–2	123 (48.4)	141 (55.7)	92 (39.7)	356 (48.2)	101 (41.7)	0.088
3	129 (50.8)	111 (43.9)	139 (59.9)	379 (51.3)	141 (58.3)	0.064
4	2 (0.8)	1 (0.4)	1 (0.4)	4 (0.5)	0	0.577
*Charlson score (%)*						
0–1	160 (63.0)	161 (63.6)	147 (63.4)	468 (63.3)	139 (57.4)	0.110
2–3	69 (27.2)	74 (29.3)	60 (25.9)	203 (27.5)	73 (52.5)	0.412
> 3	25 (9.9)	18 (7.1)	25 (10.8)	68 (9.2)	30 (12.4)	0.174
*Waiting time (days)*						
All patients	31 (24;39)	29 (21;41)	28 (20;36)	30 (21;39)	29 (21;37)	0.895
Malignant tumor	31 (23;37)	28 (19;39)	27 (20;35)	28 (21;37)	28 (21;35)	0.294
NET	27 (13;32)	32 (24;42)	34 (26;42)	30 (24;40)	38 (25;52)	0.076
Benign disease	33 (29;45)	39 (24;49)	29 (20;41)	32 (23;46)	38 (20;65)	0.134
Length of stay (days)*	10 (7;17)		10 (7;17)	10 (8;16)	10 (7;17)	11 (6;18)
30-day mortality (%)	5 (2.0)	4 (1.6)	3 (1.3)12 (1.6)	5 (2.1)	0.582	
90-days mortality (%)	14 (5.5)	13 (5.1)	12 (5.2)39 (5.3)	18 (7.4)	0.209	
In-hospital deaths (%)	5 (2.0)	4 (1.6)	3 (1.3) 12 (1.6)	5 (2.1)	0.582	

^*^In-hospital deaths excluded

**Fig. 1:**
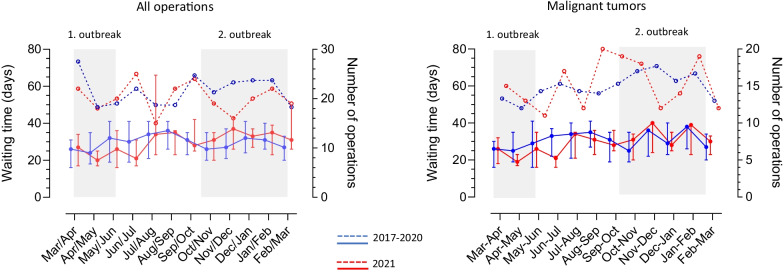
Number of operations (broken lines) and waiting time (solid lines) for pancreatic operations during COVID and the preceding three years. Number of operations from 11. March 2017 to 10. March 2020 is average of each month. Waiting time is median and quartiles

**Fig. 2:**
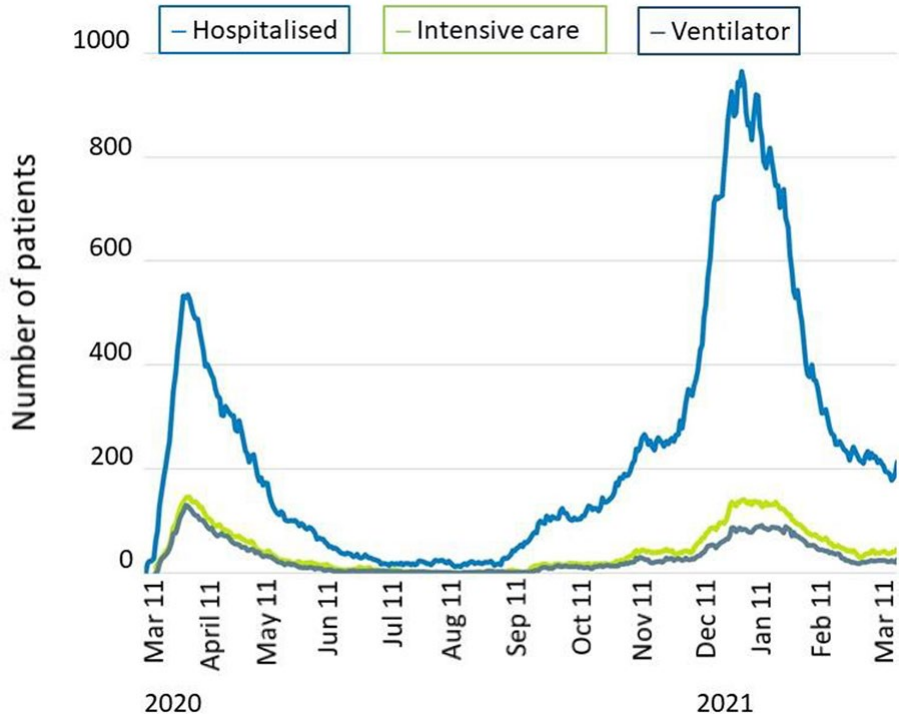
Hospitalised patients with COVID in Denmark from March 2020 to April 2020. Data from the Danish Health Authority (http://www.sst.dk)

The annual median waiting time for all operations was not extended during 2021 compared to the preceding three years, but patients with benign diseases and low-grade NET had a longer waiting time both before and during lockdown (p = 0.001). Benign tumors with planned postponement of operation were IPMN and NET WHO grade 1 and 2, while grade 3 NET and neuroendocrine carcinomas had priority like other malignant tumors. No patient had operation postponed due to COVID infection. The monthly number of operations and waiting time is shown in Fig. 1 and the number of national admissions due to COVID in Fig. 2.

During lockdown and the preceding three years as well, there were fluctuations in number of operations and waiting time. In 2021, the number of operations reached nadir in the holiday season July to August and during the peak of the second outbreak of COVID in November to December. During the second outbreak from October to March the median waiting time for operation increased to 33 (26;39) days compared to the first outbreak from March to May, where waiting time was 23 (17;33) days (p = 0.019). An extended waiting time was also seen in patients with malignant tumors during the second outbreak, median 30 (23;36) days vs. 22 (18;30) days during the first outbreak (p = 0.001). However, fluctuations in waiting time July to August and November to March were the same in the previous three years.

The frequency of the four types of operations, pancreaticoduodenectomy (Whipples’ procedure), total pancreatectomy, distal pancreatectomy, and explorative laparotomy before and during the lockdown was not significantly different, nor was the stage of malignant tumors based on pathologic examination of the resection specimens (Table 2).

**Table 2:** Pathology and treatment of patients undergoing pancreatic surgery before and during the COVID lockdown period

	Before lockdown					
	2017/2018	2018/2019	2019/2020	Total	Lockdown	*p* value
*Tumor localization (%)*						
Number	254	253	232	739	242	
Pancreas	200 (78.7)	219 (86.6)	179 (77.2)	598 (80.9)	197 (81.4)	0.925
Papilla	17 (6.7)	12 (4.7)	24 (10.3)	53 (7.2)	16 (6.6)	0.885
Common bile duct	10 (3.9)	10 (4.0)	12 (5.2)	32 (4.3)	13 (5.4)	0.483
Duodenum	27 (10.6)	12 (4.7)	17 (7.3)	56 (7.6)	16 (6.6)	0.672
*Pathology (%)*						
Adenocarcinoma	172 (85.0)	173 (68.4)	167 (72.0)	512 (69.3)	172 (71.1)	0.629
Neuroendocrine	19 (7.5)	19 (7.5)	12 (5.2)	50 (6.8)	19 (7.9)	0.564
Other malignancy	10 (3.9)	9 (3.6)	6 (2.5)	25 (3.4) 11 (4.6)	0.431	
IPMN	33 (13.0)	16 (6.3)	21 (9.1)	70 (9.5) 18 (7.4)	0.367	
Other benign	20 (7.9)	36 (14.2)	26 (11.2)	82 (11.1)	22 (9.1)	0.403
*Tumor stage Adenocarcinoma (%)*						
0	3 (1.7)	4 (2.3)	3 (1.8)	17 (3.3)	2 (1.2)	0.186
1	8 (4.7)	23 (13.3)	27 (16.2)	79 (15.4)	23 (13.3)	0.716
2	73 (42.4)	52 (30.1)	54 (32.3)	183 (35.7)	53 (30.8)	0.387
3	66 (38.4)	63 (36.4)	67 (40.1)	205 (40.0)	83 (48.3)	0.061
4	22 (12.8)	31 (17.9)	16 (9.6)	70 (13.6)	23 (13.4)	0.999
*Operation (%)*						
Whipple	135 (53.2)	97 (38.3)	115 (49.6)	347 (47.0)	98 (40.5)	0.087
Total pancreatectomy	30 (11.8)	52 (20.6)	38 (16.4)	120 (16.2)	42 (17.4)	0.691
Distal Pancreatectomy	45 (17.7)	52 (20.6)	46^a^ (19.8)	143 (19.4)	56^b^ (23.1)	0.411
Exploration	44 (17.3)	52 (20.6)	33 (14.2)	129 (17.5)	46 (19.0)	0.876
Adjuvant chemotherapy adenocarcinomas (%)	117 (68.0)	132 (76.3)	118 (70.7)	367 (71.7)	125 (72.7)	0.263

Patients operated by robotic surgery: ^a^12, ^b^26

The overall median LOS before and during lockdown was 10 (7;17) vs. 11 (16;18) days, respectively, (p = 0.966). When divided into patients who had either resection or explorative laparotomy, LOS was 12 (8;18) vs. 13 (8;20) days (p = 0.417), and 6 (4;48) days vs. 4 (4;49) days (p = 0.083), respectively.

The 30-days and 90-days postoperative mortality was 2.1% and 7.4%, respectively, during 2021 and not significantly different from the preceding three years (Table 1). The incidence of postoperative complications during lockdown was not significantly different from the preceding three years (Table 3). During lockdown, three in-patients had nosocomial infection with COVID from an infected visitor. Two of the patients were transferred to a COVID unit and were later discharged. The last patient with pre-existing chronic pulmonary disease, who had a distal pancreatic resection due to a NET, was transferred to the ICU where he died from respiratory distress.

**Table 3:** Complications to pancreatic surgery

	Before lockdown						
	2017/2018	2018/2019	2019/2020	Total	Lockdown	*p* value	
Number of operations	254	253	232	739	242		
*Clavien–Dindo (%)*							
1	33 (13.0)	21 (8.3)	19 (8.2)	73 (9.9)	22 (9.1)	0.803	
2							
3a	14 (5.5)	23 (9.1)	30 (12.2)	67 (9.1)	28 (11.6)	0.261	
3b	33 (13.0)	22 (8.7)	31 (13.4)	86 (11.6)	28 (11.6)	1.000	
4a	2 (0.8)	0	0	2 (0.3)	2 (0.8)		
4b	3 (1.2)	0	0	3 (0.4)	2 (0.8)		
5	5 (2.0)	4 (1.6)	3 (1.3)	12 (1.6)	5 (2.1)	0.582	
Surgical complications	83 (32.7)	67 (26.5)	82 (35.4)	232 (31.4)	69 (28.5)	0.423	
Wound dehiscence or infection	33 (13.0)	21 (8.3)	19 (8.2)	73 (9.9)	22 (9.1)	0.803	
Pancreatic fistula	25 (9.8)	20 (7.9)	31 (13.4)	76 (10.3)	29 (12.0)	0.473	
*Pancreaticoduodenectomy*							
Grade B	16 (6.3)	6 (2.4)	11 (4.7)	33 (4.5)	9 (3.7)	0.717	
Grade C	2 (0.8)			2 (0.3)	2 (0.8)		
*Left pancreatectomy*							
Grade B	7 (2.8)	14 (5.5)	19 (8.2)	40 (5.4)	18 (7.4)	0.271	
Grade C							
Bile fistula	11 (4.3)	12 (4.7)	15 (6.5)	38 (5.1)	11 (4.6)	0.865	
Intraabdominal abscess	8 (3.1)	9 (3.6)	10 (4.3)	27 (3.7)	10 (4.1)	0.701	
Hemorrhage	3 (1.2)	3 (1.2)	2 (0.9)	8 (1.1)	5 (2.0)		
Gastric bleeding	1 (0.3)	0	2 (0.9)	3 (0.4)	1 (0.4)		
Gastric or intestinal infarction	1 (0.3)	0	2 (0.9)	3 (0.4)	0		
Liver infarction	1 (0.3)	2 (0.8)	1 (0.4)	3 (0.4)	1 (0.4)		

Fistulas from pancreatico-jejunostomi and hepatico-jejunostomy treated with percutaneous transhepatic cholangiography assisted drainage under general anesthesia are classified as Clavien–Dindo 3b

The number of patients who had adjuvant chemotherapy after operation for adenocarcinoma was not significantly different from the previous years, but any difference in waiting time could not be evaluated.

## Discussion

Contrary to what may have been expected, there was neither asignificant difference in the waiting time nor in the treatment of patients with malignant pancreatic and periampullary tumours during the COVID lockdown compared with the preceding years.

Operations for benign diseases including IPMN and NET of low malignancy were postponed if this had an influence on the capacity to operate adenocarcinomas or other high-risk neoplasms, and this procedure was also followed before the lockdown. Our center did not receive inquiries to admit patients from other Danish regions due to lack of capacity elsewhere. Patients admitted after lockdown had the same comorbidity and ASA score than during the preceding three years. Thus, the variation in number of operations and waiting time was likely due to the patient flow.

Waiting time and number of operations would be inversely co-related, if surgical capacity was the only decisive parameter. However, this balance is disturbed by out-coming factors such as variations in patient flow, the capacity at the wards influenced by changes in LOS, and other operations than pancreatic surgery including acute surgery. Although this variation will equalize over the year, monthly fluctuations were present and with a significant peak during the second outbreak of COVID. The implementation of robotic surgery for selected cases of distal pancreatectomy from September 2019 and onwards did not influence the number of operations, neither when compared with previous years nor when corrected for more months during lockdown than the period before with robotic surgery.

Time from presentation to referral of symptomatic patients from primary into secondary care can obviously not be analyzed from our data. However, a reluctance to access healthcare with late presentation at the MDT, higher stage of malignant tumours, and a higher number of extended operations were not seen. Thus, total pancreatectomy and the incidence of stage 3 and 4 adenocarcinomas were not increased during the lockdown, so the time to referral of patients with malignant tumours had probably not been severely delayed by COVID.

**Fig. 3:**
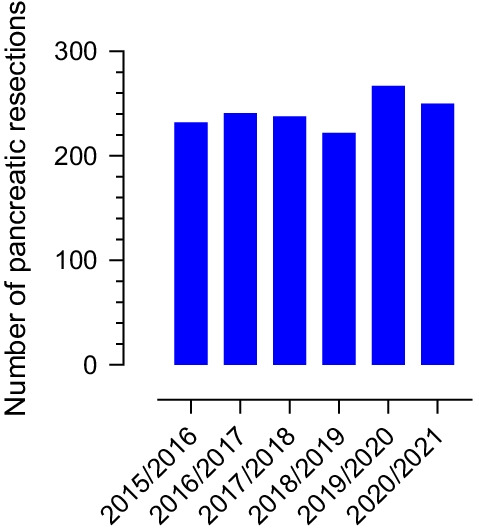
Number of resections for pancreatic and periampullary adenocarcinomas from 1. July 2015 to 30. June 2021 in Denmark. Data from the Danish Pancreatic Cancer Group (http//: www.dmsc.dk)

A Danish report found an overall reduction in the incident of all cancer diagnoses during the first three months of the pandemic compared with the previous five years [14]. However, according to data from the Danish Pancreatic Cancer Group, who runs a national register over operations and oncologic treatment, there was no deviation in the number of pancreatic operations from 1. July 2015 to 30. June 2021 that indicated any influence from COVID (Fig. 3).

When patient flow was compared with the preceding three years, the variation in the number of operations was non-significant. Both before and during lockdown there were annual fluctuations in the number of operations caused by variation in surgical capacity and in number of beds. There were two major peaks of hospital admissions in Denmark due to COVID (Fig. 2), the first from March to July 2020 and the second from September to April 2021, most cases in the capital region, but this dot seem to have an impact on the number of pancreatic operations in our department. The decreasing number of operations in July and August with an increase in waiting time was coincident with the holiday session. During the second and longest wave of COVID admissions from October 2020 to February 2021 there was a fall in number of operations from October to December with an increase in waiting time for operation. The drop in the number of operations was smaller during the preceding three years but waiting time showed a similar tendency, most notably for patients with malignant tumours, and so the timely access to operation for malign pancreatic tumors during lockdown was like the preceding years.

Before lockdown close to 30% of our patients operated for adenocarcinoma did not receive adjuvant treatment, either due to low performance or because patients opted out of oncologic treatment. These figures were not different during lockdown. Because chemotherapeutic treatment first starts when patients’ clinical condition is favorable, although not later than 12 weeks from operation, it was not possible to distinguish between waiting time due to COVID or due to patients’ clinical condition.

From the beginning of the outbreak, several countries made plans for necessary surgical treatment based on the availability of medical resources [15, 16]. For elective surgery of non-malignant diseases this may have been an easy task contrary to the reduction of oncologic surgery [17–20]. Preoperative chemotherapy in upfront resectable tumors with delayed surgery was proposed [21, 22]. Most notable, national clinical guidelines issued for the United Kingdom for the management of essential cancer surgery for adults during the coronavirus pandemic asserted that pancreatic surgery might wait up to three months [23]. This was not supported by a subsequent publication from ESMO based on observational studies in England from 2013–2017 with modelled data for three and six months delay of surgery. The study concluded that a 12-weeks delay might reduce overall survival for most cancer patients and especially patients with pancreatic cancer. [24], and this was also supported by a British Swiss meta-analysis [25].

Like other countries Danish health service operates with a hub-and-spoke organizational model with highly specialized hospitals supplemented by secondary hospitals with more limited arrays of medical services. This model was not challenged during lockdown, nor was it necessary to establish “hot” and “cold” hospitals [4], but each hospital set up isolated units for COVID patients. Centralization of pancreatic surgery to four specialized hospitals in Denmark may have proven beneficial, not only to lower postoperative morbidity and mortality but also in dealing with complication to COVID.

Denmark is one of the countries in Western Europe with the lowest number of hospital beds pr. 100.000 inhabitants (Table 4). This is the result of a large reconstruction of the hospital service during the last 40 years with focus on fast track surgery, more out-patient treatment, centralization and merging of units to save costs. Denmark has no major private health sector with hospitals to assist if the access to cancer treatment is severely impaired. During the pandemic, the hospital capacity gave rise to serious concern among the health authorities. This concern turned out to be unfounded but only thanks to a relatively small number of infected and hospitalized patients and deaths compared to other countries. Nevertheless, the relatively small number of hospitalized patients due to COVID may have saved the Danish health system from a break down and cancellation of cancer operations in a scale that was seen in several other European countries.

**Table 4:** Health spending, hospital beds, COVID cases and deaths 16 March 2020 to 15 March 2021

	Health spending	Hospital beds	Cases	Deaths
	USD per capita	Per 100.000	Per 100.000	Per 100.000
Spain	3.600	300	6.831	162
Italy	3.819	320	5.565	174
France	5.274	580	6.047	136
United Kingdom	5.268	240	6.395	188
Denmark	5.478	260	3.795	41
Ireland	5.604	290	3.443	70
Sweden	5.754	214	7.206	129
Austria	5.899	720	5.672	101
Germany	6.731	790	4.035	126

Health spending and hospital beds: OECD data 2020 (http://www.data.oecd.org)

COVID cases and deaths: WHO (http://www.covid19.who.int)

We studied a well-defined patient group from a high-volume center with well established guidelines for pancreatic surgery, which enabled us to analyze the impact of the COVID lockdown on surgical treatment compared with historical data. Moreover, the complete data registration makes results valid within the chosen parameters. However, the study also has limitations due to insufficient information such as patients’ contact to the primary care, as the risk of infection may have prevented especially frail patients contact their general practitioner. A number of factors in our clinic such as variation in surgical capacity due to other operations than pancreatic surgery, cancellation of activity owing to changing capacity at the wards and redeployment of staff to COVID sections may have an undefined impact on the results. These factors can hardly be incorporated in the analysis and their impact remains unknown. The retrospective design of the study engenders the usual caveats, but due to the number of patients and the consistent registration of data, this is not expected to influence our results.

## Conclusion

The lockdown during COVID did not have a measurable influence on pancreatic surgery in our clinic. This was clear from all measured parameters. The limitation of the surgical treatment depends on the available public resources, which were sufficient, as long as the number of admissions due to the basic reproductive rate of COVID in Denmark was lower compared to other European countries.

## Data Availability

The datasets used and/or analysed during the current study are available from the corresponding author on reasonable request.

## Abbreviations

ASA: The American Society of Anesthesiologists
ICU: Intensive care unit
IPMN: Intraductal papillary mucinous neoplasia
LOS: Length of stay
MDT: Multidisciplinary team conference
NET: Neuroendocrine tumour
PDAC: Pancreatic ductal adenocarcinoma
